# Sex Difference of Effect of *Sophora flavescens* on Gut Microbiota in Rats

**DOI:** 10.1155/2022/4552904

**Published:** 2022-03-15

**Authors:** Xueqing Duan, Xin Xie, Chen Zhu, Zhixuan Duan, Rui Chen, Jun Xu, Qi Zhang, Qi Yu, Weiyi Tian

**Affiliations:** Guizhou University of Traditional Chinese Medicine, Gui Yang 550025, China

## Abstract

**Objective:**

By observing the sex difference of the gut microbiota in rats and the influence of *Sophora flavescens* (*S. flavescens*) on the gut microbiota in rats of different genders, it is hoped that it can provide reference materials for the rational use of *S. flavescens* in clinical practice.

**Method:**

Taking samples of the jejunum (containing intestinal contents) and feces of 8-week-old rats, and detecting the composition of gut microbiota of females and males by 16S rRNA sequencing technology; At the same time, 8-week-old rats were gavaged with different doses of *S. flavescens* decoction, and the duodenum, jejunum, ileum, and colon (including the intestinal contents) samples were collected at 1, 2, and 3 weeks, using polymerase chain reaction-denaturing gradient gel electrophoresis (PCR-DGGE) technology and real-time fluorescent quantitative PCR (qRT-PCR) technology to observe the changes in the structure and the quantitative changes of 4 major intestinal dominant bacteria *Enterococcus*, *Bacteriodes*, *Lactobacillus*, and *Clostridium* in each intestinal segment, respectively.

**Result:**

(1) The gut microbiota of normal rats without administration also had obvious gender differences; (2) *S. flavescens* significantly affects the composition of gut microbiota, and in different intestinal segments, this effect was different between genders under different dosages and different continuous administration times.

**Conclusion:**

The effect of *S. flavescens* on the gut microbiota of rats had gender differences.

## 1. Introduction

The human intestine contains extremely complex types of bacteria, about 500–1000 species, and these bacteria are collectively refered to as the gut microbiota [1]. At present, it has been found that the intestine contains at least 9 bacterial phyla, among which *Enterococcus, Lactobacillus*, *Bacteriodes*, and *Clostridium* are the main dominant bacteria genera in the intestinal tract [2, 3]. The gut microbiota is closely related to the body, and it plays many important functions, such as material metabolism, biosynthesis, protection, and nerve function [4]. The composition and structure of the normal human intestinal flora remain relatively stable. Under the influence of drugs, diet, and living habits, the structure and function of the gut microbiota change, thus promoting intestinal inflammation, metabolic diseases, and immunity, leading to the occurrence and development of diseases such as sexual diseases, neurological diseases, and tumors [5]. Clinical studies have shown that people of different genders have dimorphisms in disease prevalence, age of disease, diagnosis and treatment methods, etc. and are associated with sex hormone, gene, and behavior differences. For example, age-adjusted incidence rate (AIR) is higher in men for diseases such as tumors and respiratory diseases, and higher in women for diseases such as endocrine and metabolic disorders [6]. Similarly, the structure of gut microbiota is potentially sex-dependent and the differences in the structure of the gut microbiota between sexes are closely related to the level of sex hormones [7–10]. Takagi et al. [11] found that there were significant differences in the structure of the intestinal flora between different sexes, and the relative abundance of *Prevotella*, *Macromonas*, *Fusobacterium*, and *Macrococcus* in the digestive tract of males was higher, while that of females relative abundance of *Bifidobacterium*, *Rumenococcus*, and *Akkermania* was higher in the digestive tract. Although gender differences are closely related to the gut microbiota and diseases, up to now, the specific mechanism of the interaction between the three is unclear, and there are no clinical cases of disease prevention and treatment targeting gender-dependent gut microbiota.

Traditional Chinese medicine (TCM)) is a medicine for preventing and curing diseases under the guidance of a unique theory. It is also called a natural medicine and is widely used in clinical practice. Oral administration is the main way of TCM administration, which is digested and absorbed by the human body through the gastrointestinal tract. Therefore, the relationship between TCM and gut microflora has attracted people's attention. Modern studies have found that the main interaction modes between TCM and gut microflora are as follows: TCM regulates intestinal flora structure, TCM regulates metabolism of gut microflora, and the components of TCM plays a role in gut microflora transformation and metabolism [12–14]. Bitter-cold Chinese medicines (such as *Rhizoma coptidis*, *Pueraria lobata*, and *S. flavescens*) have anti-inflammatory, antibacterial, antiviral, and antitumor effects, and they also have the effect of regulating gut microflora [15, 16]. Yao et al. [17] found that berberine, the main component of *Rhizoma coptidis*, could increase the abundance of the phylum *Bacteroides* in the intestinal tract of type 2 diabetic rats, and reduced the abundance of the phylum *Proteobacteria* and *Vertexia*, while at the family level, it increased the abundance of *Lactobacillaceae*. Puerarin, the active ingredient of *Pueraria lobata*, can alleviate obesity induced by a high-fat diet by upregulating the abundance of *Akkermansia muciniphila* in mice [18]. TCM *S. flavescens* is the dried root of *S. flavescens Ait*, which is the *Sophora* plant of the leguminous family. *S. flavescens* is “cold in nature” and “bitter in taste.” Its common pharmacological effects are anti-inflammatory, antitumor, antidiabetic, analgesic, antipruritic, etc. The main active ingredients are alkaloids, flavonoids, and polysaccharides [19, 20]. However, there are few reports on gender differences in the use of TCM and the interaction between intestinal microflora and *S. flavescens*, and the specific mechanism is not yet clear.

In this study, we observed the gender difference of gut microflora in rats and the changes in intestinal flora diversity and the abundance changes of dominant bacteria in each intestinal segment after *S. flavescens* intervention in both female and male rats so as to provide basic data for the clinical use of *S. flavescens* and on the difference in the use of TCM between the sexes.

## 2. Materials and Methods

### 2.1. Animal

150 SPF (specific pathogen-free) SD (Sprague–Dawley) rats, male and female, were purchased from Chongqing Tengxin Biotechnology Co., Ltd., experimental animal production license no. SCXK (Jun) 2012-0011. The animals were raised in the experimental Animal Center of Guizhou Medical University, license number SYXK (Guizhou) 2018-0001. The feeding temperature and humidity were (24 ± 2)°C and 50%–70%, respectively, and (12 h: 12 h) day and night intermittent lighting, free eating, and drinking water. All animal-related operations involved in this study have been approved by the Animal Ethics Committee of Guizhou Medical University (approval no. 1503018).

### 2.2. Drugs and Reagents

*S. flavescens* was purchased from Beijing Tongren Tang Guiyang drugstore and was identified as genuine by professor Ying Zhou, an expert of crude medicine in the Guizhou University of Traditional Chinese Medicine. For the preparation method of *S*. *flavescens* decoction, refer to the method reported by Duan et al. [21]. The antibiotic used in this study was *Lincomycin* injection, purchased from Henan Tianfang Pharmaceutical Co., Ltd. (batch no. 070120049). The kits used in this study are as follows: DNA Extraction Kit (Beijing Kangrunchengye Biotechnology Co., Ltd., batch no. B027008019), Total RNA Extraction Kit (Omega, lot no. R6828-02), Complementary deoxyribonucleic Acid (cDNA) Reverse Transcription Kit (Sigma, lot 0000123564), and 2 × TransStartTM SYBR Top Green qPCR SuperMix (Beijing Kang Wei Century Biotechnology Co., Ltd., batch no. CW0760).

### 2.3. Grouping, Administration, and Sampling

SD rats were randomly divided into a normal group (Ctr), an antibiotic group (Ant), and a *S. flavescens* group, among which the *S. flavescens* component was high- (7.5 g/kg; Hig), medium- (1.5 g/kg; Med), and low- (0.3 g/kg; Low) dose groups [22]. Each group had 18 animals (at least 9 males and 9 females), and the antibiotic was *Lincomycin White*. For related administration method, dosage, duration of drug, and methods of obtaining animal tissue and fecal samples, refer to the method reported by Duan et al. [21].

### 2.4. 16S Microbiota Sequencing and Analysis

Fecal and jejunum microbiological samples were collected from the normal group rats (10 males and 10 females). The V4 region of the 16S rRNA gene was amplified with barcoded-fusion primers, and paired-end 151 base-pair reads were sequenced on the Illumina MiSeq instrument as previously described [23]. The 16S microbiota sequencing and analysis of the relevant samples in this experiment was performed by Shanghai Zhijiang Bioengineering Co., Ltd.

### 2.5. PCR-DGGE Analysis

Fecal microbiota samples were collected from the rats of the normal group, antibiotic group (Ant), and *S. flavescens* group. Total bacterial DNA was extracted from the fecal samples by the kit method. Both female and male rats, primers, and methods of PCR-DGGE analysis for fecal microbiota referred to the method reported by Wu et al. [24].

### 2.6. qRT-PCR Analysis

The duodenum, jejunum, ileum, and colon (including the intestinal contents) samples were collected from the rats of the normal group, antibiotic group (Ant), and *S. flavescens* group. Total bacterial RNA was extracted from the fecal samples by the kit method. Both female and male rats, primers, and methods of qRT-PCR analysis for 4 dominant gut microbiota referred to the method reported by Duan et al. [21].

### 2.7. Statistical Analysis

In the study, the SPSS 22.0 software was used for statistical analysis of the data. The measurement data were shown as means ± s.e.m or means ± s.d, and Mann–Whitney *U* test or Student's *t*-tests were used to generate the *P* values. Mann–Whitney *U* test or *t*-test: ^*∗*^, *P* < 0.05; ^*∗∗*^, *P* < 0.01.

## 3. Results

### 3.1. Gender-Specific Gut Microbiota Composition in Rats

**Figure 1:**
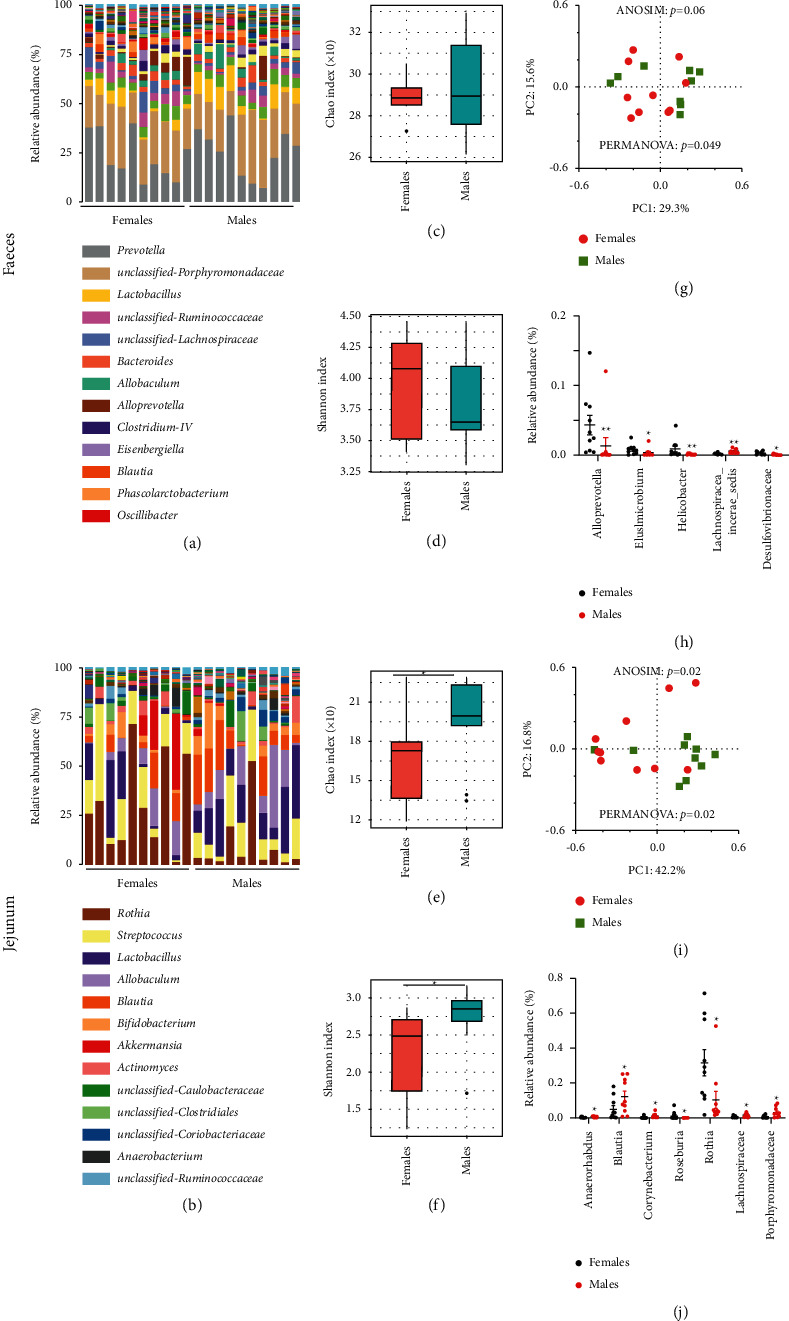
Sex differences of the gut microbiota composition in SD rats. (a, b) The relative abundance per individual for fecal and jejunal samples from female or male rats, shown for the bacterial taxonomic rank of family or genus. (c–f) Within group mean *α* diversity. (g, i) Principal coordinate analysis (PCoA) plot of Bray–Curtis distances, analysis of similarity (ANOSIM) ,and permutational multivariate analysis of variance (PERMANOVA) of the microbial communities of fecal or jejunal samples between males and females. (h, j) Relative abundance distributions per group for the taxa (at family or genus level) in fecal or jejunum samples between males and females. *n* = 10 per group, *P* values from Mann–Whitney *U* test.

In order to assess whether the microbiome is sex-related, we compared the bacterial composition of fecal and jejunal samples from female or male rats. we found significant sex differences in gut microbiota composition, especially in the jejunum (Figures 1(a) and 1(b)). In addition, jejunal samples rather than fecal samples varied in gut microbial *α*-diversity (bacterial diversity, or bacterial richness and evenness, within each group) between sexes, as quantified with the Chao and Shannon diversity index (minimum of seven individuals per sex for Mann–Whitney *U* test, MWU test; Figures 1(c)–1(f)). The principal coordinate analysis (PCoA) plot of Bray–Curtis distances of jejunal samples rather than fecal samples also showed clear patterns differentiating samples from males and females. We used analysis of similarity (ANOSIM) and permutational multivariate analysis of covariance (PERMANOVA) to test whether a matrix of major PCoA axes was dependent on sex, especially in the jejunum samples. (Figures 1(g) and 1(i); Tables S1 and S2).

To determine the basis for this variation, we identified specific taxa (at phylum, family, or genus level) that changed with sex (Figure S1; Figures 1(h) and 1(j); Table S3). At the phylum level, the abundance of *Elusimicrobia* of fecal samples in male rats was significantly higher than that in females, while the abundance of *Firmicutes* of jejunum samples in male rats was significantly higher than that in females (Figure S1). Moreover, in terms of fecal samples, the abundance of genera including *Alloprevotella* and *Helicobacter*, and family Desulfovibrionaceae in female rats was significantly higher than that in males, but the abundance of genera including *Elusimicrobium* and *Lachnospiracea_incertae_sedis* in males was significantly higher than that in females (Figure 1(h); Table S3). For the jejunum samples, the abundance of genus *Roseburia* and *Rothia* in female rats was significantly higher than that in males, but the abundance of genera including *Anaerorhabdus*, *Blautia*, *Corynebacterium*, *family* Lachnospiraceae, *and family* Coriobacteriaceae in male rats was significantly higher than that in females (Figure 1(j); Table S3).

In conclusion, the above results suggested that the gut microbiota composition has significant gender specificity in SD rats, especially in the jejunum.

### 3.2. Gender Differences in the Influence of *S. flavescens* on the Microbial Composition of Various Intestinal Segments

In order to investigate the effects of *S. flavescens* on the intestinal microflora, the PCR-DGGE method was used to analyze the changes of intestinal microflora composition in each group after 1, 2, and 3 weeks of continuous administration.

**Figure 2:**
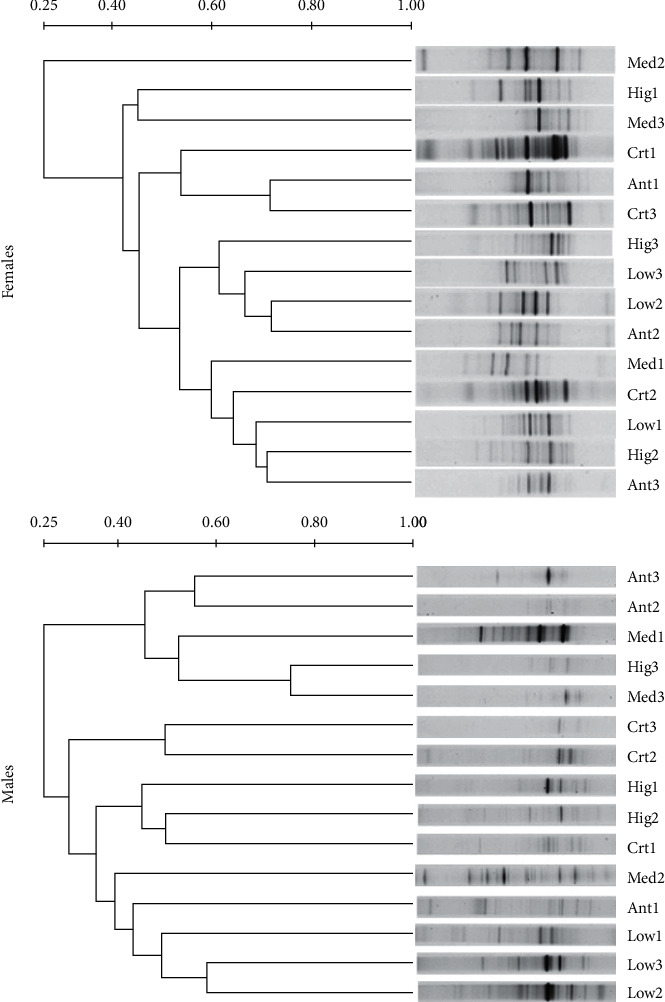
UPGAMA tree analysis of duodenal microflora from different gender rats among different groups. Ctr, Low, Med, Hig, and Ant represent the normal group, low-, medium-, high-dose groups of *S. flavescens* and antibiotic group, respectively. 1, 2, and 3 represent the rats for 1, 2, and 3 weeks of continuous drug intervention.

In the duodenum of female rats, compared with other groups, the DNA band similarity of microbiota samples was higher between the Ant group and Ctr group after 1 weeks of continuous administration (>60%). DNA band similarity of microbiota samples was higher (>60%) between the Ant group and Low group that were continuously administered for 2 weeks, respectively, and DNA band similarity of microbiota samples was higher between the Ant group and Hig group after 3 weeks of continuous administration. For males, however, compared with other groups, DNA band similarity of microbiota samples was higher (>70%) between the Hig group and Mid group after 3 weeks of continuous administration in the duodenum, while the similarity among the other groups was low. In addition, there were significant differences in DNA band similarity of duodenal microbe samples between sexes at the same dose and for the same duration of continuous administration (Figure 2).

**Figure 3:**
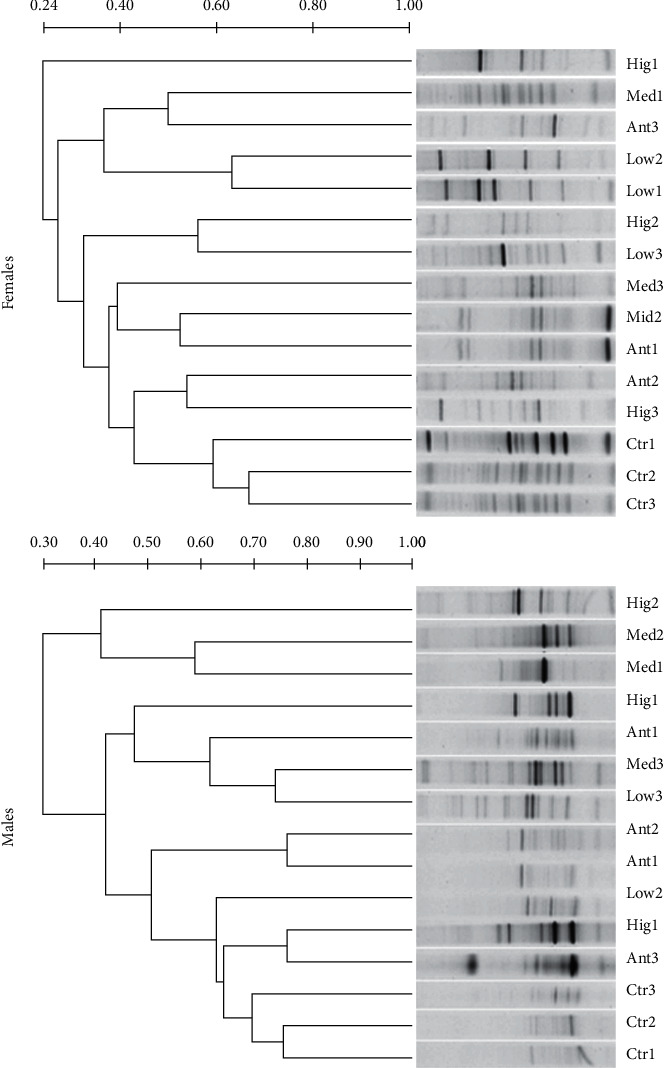
UPGAMA tree analysis of jejunal microflora from different gender rats among different groups. Ctr, Low, Med, Hig, and Ant represent normal group, low-, medium-, and high-dose groups of *S. flavescens* and antibiotic group, respectively. 1, 2, and 3 represent the rats for 1, 2, and 3 weeks of continuous drug intervention.

In the jejunum of female rats, compared with other groups, the DNA band of microbiota samples in the Ctr group had certain similarity between 1, 2, and 3 weeks of continuous administration, and the DNA band similarity of microbiota samples between 2 and 3 weeks of continuous administration was higher (>60%). The DNA band similarity of microbiota samples in the Low group was higher (>60%) between 1 and 2 weeks of continuous administration. For males, compared with other groups, however, the DNA bands of jejunum microbe samples in the Ant group between 1 and 2 weeks of continuous administration were highly similar (>70%), and the DNA bands of jejunum microbe samples between the Hig group after 1 week of continuous administration and Ant group after 3 weeks of continuous administration were highly similar (>70%). The DNA bands of jejunum microbe samples in the Ctr group between 1, 2, and 3 weeks of continuous administration had high similarity (>60%), and the DNA band similarity of microbiota samples between 1 and 2 weeks of continuous administration was higher (>70%). The similarity of DNA bands of jejunum microbial samples in the Med group and Low group after 3 weeks of continuous administration was higher (>70%), while the similarity among the other groups was low. In addition, there were significant differences in the DNA band similarity of jejunum microbial samples between sexes at the same dose and for the same duration of continuous administration (Figure 3).

**Figure 4:**
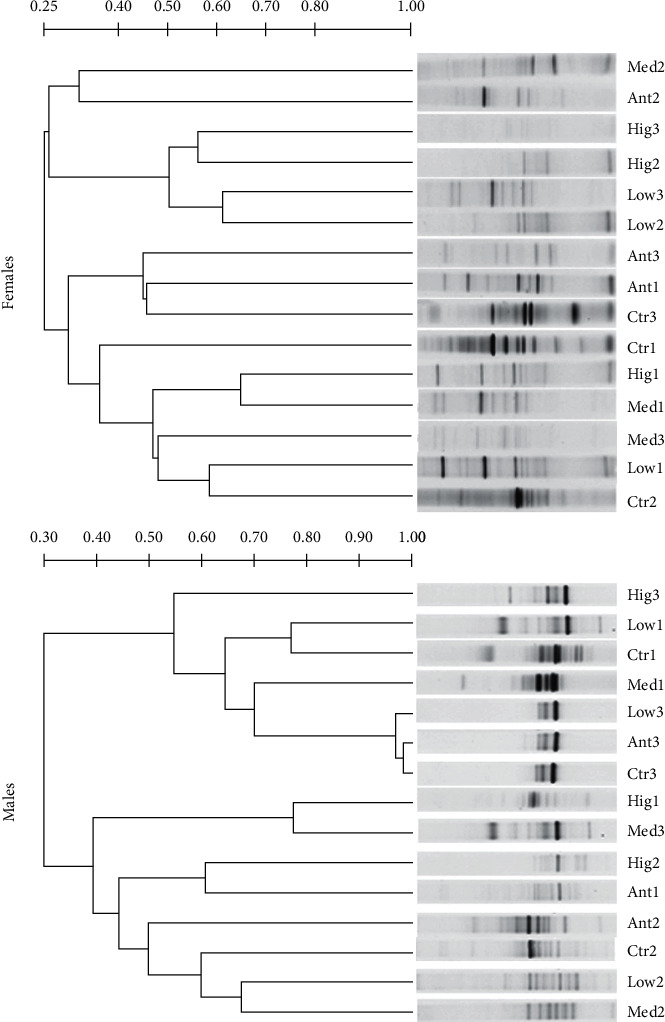
UPGAMA tree analysis of ileac microflora from different gender rats among different groups. Ctr, Low, Med, Hig, and Ant represent the normal group, low-, medium-, high-dose groups of *S. flavescens*, and antibiotic group, respectively. 1, 2, and 3 represent the rats for 1, 2, and 3 weeks of continuous drug intervention.

In the ileum of female rats, compared with other groups, the DNA band of microbiota samples in the Low group had a certain similarity (>60%) between 2 and 3 weeks of continuous administration; the DNA bands of the microbial samples between the Med group and Hig group had a certain similarity (>60%) after 1 week of continuous administration. For males, compared with other groups, however, the similarity of DNA bands of microbial samples was extremely high (>90%) between the Low group, Ant group, and Ctr group of *S. flavescens* after continuous administration for 3 weeks. The DNA bands of microbial samples had a certain similarity (>60%) between the Med group after 1 week of continuous administration and Low group, Ant group, and Ctr group that were continuously administered for 3 weeks, respectively. The DNA bands of microbial samples had high similarity (>70%) between the Hig group after 1 week of continuous administration and the Med group that were continuously administered for 3 weeks. The DNA bands of microbial samples between the Low group and Ctr group after 1 week of continuous administration had a high similarity (>70%). The DNA bands of microbial samples between the Low group and Med group after 2 weeks of continuous administration had a certain similarity (>60%), while the similarity among the other groups was low. In addition, there were significant differences in the DNA band similarity of ileac microflora samples between sexes at the same dose and for the same duration of continuous administration (Figure 4).

**Figure 5:**
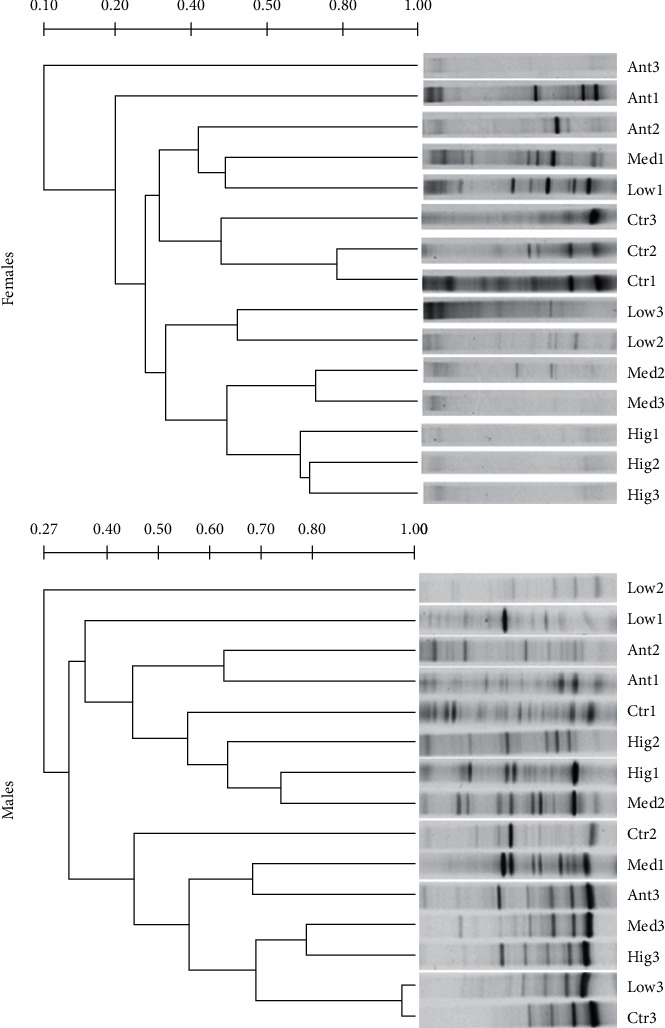
UPGAMA tree analysis of colonic microflora from different gender rats among different groups. Ctr, Low, Med, Hig, and Ant represent the normal group, low-, medium-, high-dose groups of *S. flavescens* and antibiotic group, respectively. 1, 2, and 3 represent the rats for 1, 2, and 3 weeks of continuous drug intervention.

In the colon of female rats, compared with other groups, the DNA band of microbiota samples in the Ctr group had a certain similarity (>80%) between 1 and 2 weeks of continuous administration. There was a certain similarity (>60%) in the DNA band similarity of microbiota samples derived from the Med group between 2 and 3 weeks of continuous administration; the DNA band of microbiota samples in the Hig group had a certain similarity (>60%) between 1, 2, and 3 weeks of continuous administration. For males, compared with other groups, however, the DNA band similarity of microbiota samples was very high (>90%) between the Ctr group and Low group after 3 weeks of continuous administration. There was a high similarity (>70%) in the DNA bands of the microbe samples between the Med group and Hig group after 3 weeks of continuous administration. The DNA bands of microbial samples had a high similarity (>70%) between the Med group after 2 weeks of continuous administration and Hig group that was administered for 1 week. There was a certain similarity (>60%) in the DNA bands of microbial samples between the Med group after 1 weeks of continuous administration and Ant group that was administered for 3 weeks, while the similarity among the other groups was low. In addition, there were significant differences in DNA band similarity of colonic microflora samples between sexes at the same dose and for the same duration of continuous administration (Figure 5).

In summary, above results suggested that (1) the microflora structure of female and male rats changed with the prolongation of feeding time; and (2) *S. flavescens* could affect the microflora of SD rats, and the effect had obvious dose-time (dosing dose and duration of dosing) regularity and gender difference.

### 3.3. Gender Differences in the Effects of *S. flavescens* on 4 Major Intestinal Dominant Bacteria such as *Enterococcus*, *Bacteriodes*, *Lactobacillus*, and *Clostridium* in Each Intestinal Segment, Respectively

To further study the gender difference in the effect of *S. flavescens* on the gut microbiota, we analyzed the quantity changes of *Enterococcus*, *Bacteriodes*, *Lactobacillus*, and *Clostridium* in each intestinal segment after continuous administration of *S. flavescens* by RT-PCR, respectively.

**Figure 6:**
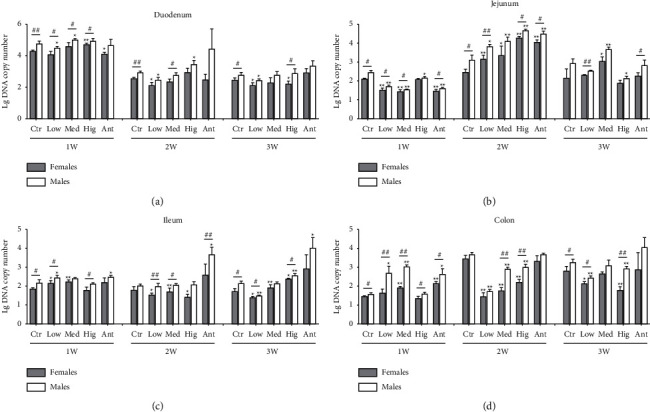
Effect of *S. flavescens* on the number of *Enterococcus* in various segments of rats with different genders. (a–d) Changes in the number of *Enterococcus* in duodenum, jejunum, ileum, and colon after 1, 2, and 3 weeks of drug intervention, respectively. Ctr, Low, Med, Hig, and Ant represent the normal group, low-, medium-, and high-dose groups of *S. flavescens* and antibiotic group, respectively. 1, 2, and 3 represent the rats for 1, 2, and 3 weeks of continuous drug intervention. The data were shown as means ± s.e.m, *n* ≥ 3, Student's *t*-tests were used to generate the *P* values. *t*-test: ^*∗*^, *P* < 0.05; ^*∗∗*^, *P* < 0.01 versus Ctr; ^#^*P* < 0.05; ^##^*P* < 0.01 females versus males.

For *Enterococcus*, in duodenum: after continuous drug intervention, compared with the Ctr group, the number of enterococcus both in female and male rats in the Low group was significantly reduced after continuous drug intervention; The number of *Enterococcus* both in males from the Ant group and females from the Med group did not change significantly. The number of *Enterococcus* in females from the Hig group increased first and then decreased. The number of *Enterococcus* in females from the Ant group decreased first and then recovered. The number of *Enterococcus* in males from the Med group or Hig group first increased and then recovered. Interestingly, except for the Ant group after 1 week of drug intervention, Low group, Hig group, and Ant group after 2 weeks of drug intervention, and Low group, Med group, and Ant group after 3 weeks of drug intervention, the number of *Enterococcus* in male rats in the other groups was significantly higher than that in females (Figure 6(a). In jejunum: after continuous drug intervention, compared with the Ctr group, the number of *Enterococcus* in female rats from the Low group, females and males from the Ant group decreased first, then increased, and finally returned to normal; The number of *Enterococcus* both in females and males from the Med group decreased first and then increased, while the number of *Enterococcus* both in females and males from the Hig group increased first and then returned to normal. Additionally, the number of *Enterococcus* in male rats was significantly higher than that in females in the other groups, except that there was no difference between males and females in the Hig group after 1 and 3 weeks of drug intervention, in the Med group after 2 and 3 weeks of drug intervention and in the Ant group after 3 weeks of drug intervention (Figure 6(b)). In ileum: after continuous drug intervention, compared with the Ctr group, the number of *Enterococcus* both in female and male rats from the Low group and females from the Med group increased first and then decreased. The number of *Enterococcus* in male rats from the Hig group or Ant group was significantly increased, while the number of *Enterococcus* in females from the Hig group was firstly decreased and then increased. Moreover, in the other groups, the number of *Enterococcus* in male rats was significantly higher than that in females, except that there was no difference in the number of *Enterococcus* between males and females in the Med group and Ant group after 1 and 3 weeks of drug intervention, and in the Ctr group and Hig group after 2 weeks of intervention (Figure 6(c)). In colon: after continuous drug intervention, compared with the Ctr group, the number of *Enterococcus* both in female rats from the Low group and Hig group in females and males was significantly decreased. In the Med group, the number of *Enterococcus* both in males and females firstly increased, then decreased, and finally returned to normal. Furthermore, in the other groups, the number of *Enterococcus* in male rats was significantly higher than that in females, except for the Ctr group, Low group, and Ant group after 2 week of drug intervention and Med group and Ant group after 3 weeks of drug intervention (Figure 6(d)).

**Figure 7:**
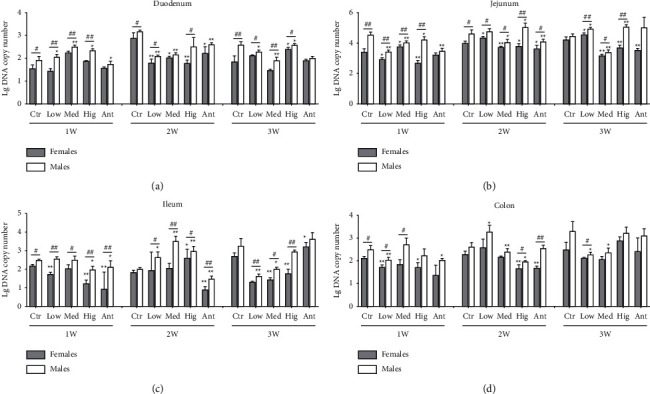
Effect of *S. flavescens* on the number of *Bacteriodes*, in various segments of rats with different genders. (a–d) Changes in the number of *Bacteriodes* in the duodenum, jejunum, ileum, and colon after 1, 2, and 3 weeks of drug intervention, respectively. Ctr, Low, Med, Hig, and Ant represent the normal group, low-, medium-, high-dose groups of *S. flavescens*, and antibiotic group, respectively. 1, 2, and 3 represent the rats for 1, 2, and 3 weeks of continuous drug intervention. The data were shown as means ± s.e.m, *n* ≥ 3, Student's *t*-tests were used to generate the *P* values. *t*-test: ^*∗*^, *P* < 0.05; ^*∗∗*^, *P* < 0.01versus Ctr; ^#^*P* < 0.05; ^##^*P* < 0.01 females versus males.

For *Bacteriodes*, in duodenum: after continuous drug intervention, compared with the Ctr group, the number of *Bacteriodes* of female rats in the Low group, Med group, and Ant group decreased first and then returned to normal. The number of *Bacteriodes* in female rats in the Hig group was first decreased and then increased, while the number of *Bacteriodes* in males in the Low group, Med group, and Hig group was first increased and then decreased. Interestingly, in the other groups, the number of *Bacteriodes* in male rats was significantly higher than that in females, except for the Ant group after 1 and 3 week of drug intervention, Med group and Ant group after 2 weeks of drug intervention (Figure 7(a)). In jejunum: after continuous drug intervention, compared with the Ctr group, The number of *Bacteriodes* both in female rats from the Low group and males from the Hig group decreased first and then increased; The number of *Bacteriodes* in female rats from the Med group was firstly increased and then decreased, while the number of *Bacteriodes* both in females from the Hig group or Ant group and males from the Med group was significantly decreased. Moreover, in the other groups, the number of *Bacteriodes* in male rats was significantly higher than that in females, except that there was no difference in the number of *Bacteriodes* between male and female rats in the Ant group after 1 and 3 weeks of drug intervention and in the Ctr group after 3 weeks of drug intervention (Figure 7(b)). In ileum: after continuous drug intervention, compared with the Ctr group, the number of *Bacteroides* both in female or male rats from the Low group and males from the Hig group first decreased and then increased. In the Med group, the number of *Bacteroides* in female rats increased first and then decreased. The number of *Bacteroides* both in female rats of the Hig group, Ant group and males of the Med group was significantly decreased, while the number of *Bacteroides* in males of the Ant group was first decreased and then returned to normal. The number of *Bacteroides* in female rats of the Low group and males of the Ant group was first decreased and then returned to normal. The number of *Bacteroides* was first decreased and then increased both in male rats of the Hig group and females of the Ant group, which was inverse in male rats from the Low group and Med group. The number of *Bacteroides* in female rats of the Hig group was first decreased, then increased and finally decreased, while the number of *Bacteroides* in female rats of the Med group was significantly decreased. In addition, except the Ctr group after 2 or 3 weeks of drug intervention and Ant group after 3 weeks of intervention, in other groups, the number of *Bacteroides* in male rats was significantly higher than that in females (Figure 7(c)). In colon: after continuous drug intervention, compared with the Ctr group, The number of *Bacteroides* both in female rats of the Low group, Hig group, and Ant group and males of the Hig group decreased first and then returned to normal. In the Low group, the number of *Bacteroides* in male rats first decreased, then increased, and then decreased, while the number of *Bacteroides* in males from the Med group was significantly reduced. Besides, in terms of sex, in the Ctr group, Low group, and Med group, the number of *Bacteroides* in male rats was significantly higher than that in females after 1 week of drug intervention (Figure 7(d)).

**Figure 8:**
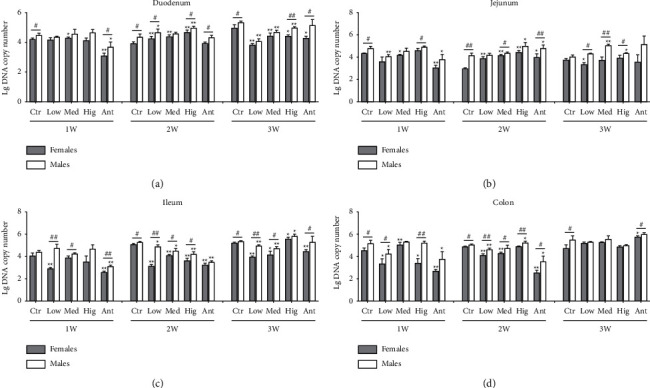
Effect of *S. flavescens* on the number of *Lactobacillus*, in various segments of rats with different genders. (a–d) Changes in the number of *Lactobacillus* in the duodenum, jejunum, ileum, and colon after 1, 2, and 3 weeks of drug intervention, respectively. Ctr, Low, Med, Hig, and Ant represent the normal group, low-, medium-, and high-dose groups of *S. flavescens* and antibiotic group, respectively. 1, 2, and 3 represent the rats for 1, 2, and 3 weeks of continuous drug intervention. The data are shown as means ± s.e.m, *n* ≥ 3, and Student's *t*-tests were used to generate the *P* values. *t*-test: ^*∗*^, *P* < 0.05; ^*∗∗*^, *P* < 0.01 versus Ctr; ^#^*P* < 0.05; ^##^*P* < 0.01 females versus males.

For *Lactobacillus*, in duodenum: after continuous drug intervention, compared with the Ctr group, the number of *Lactobacillus* both in female and male rats of the Low group and Hig group and females of the Med group increased first and then decreased. Except for the Low group after 1 and 3 weeks of drug intervention, Med group after 1, 2, and 3 weeks of intervention, and Hig group after 1 week of intervention, in other groups, the number of *Lactobacillus* in male rats was significantly higher than that in females (Figure 8(a)). In jejunum: after continuous drug intervention, compared with the Ctr group, in the Low group, the number of *Lactobacillus* increased first and then decreased in female rats but decreased first and then returned to normal in males. The number of *Lactobacillus* in male rats was significantly increased in the Med group and Hig group. Additionally, after 1 week of drug intervention, the number of *Lactobacillus* in male rats was significantly higher than that in females both in the Ctr group and Hig group. After 3 weeks of intervention, the number of *Lactobacillus* in male rats was significantly higher than that in females in the Low group, Med group, and Hig group (Figure 8(b)). In ileum: after continuous drug intervention, compared with the Ctr group, the number of *Lactobacillus* both in female and male rats in the Low group and Med group and females in the Ant group was significantly decreased, while the number of *Lactobacillus* of female and male rats in the Hig group decreased first and then increased. Moreover, except for the Ctr group and Hig group after 1 week of drug intervention, the Ant group after 2 weeks of intervention, and the Hig group and Ant group after 3 weeks of drug intervention, in other groups, the number of *Lactobacillus* in male rats was significantly higher than that in females (Figure 8(c)). In colon: after continuous drug intervention, compared with the Ctr group, the number of *Lactobacillus* both in female rats from the Low group or Hig group and males from the Low group or Ant group decreased first and then returned to normal. In the Med group, the number of *Lactobacillus* in female rats first increased, then decreased, and finally returned to normal in female rats, while there was no significant change in male rats. Interestingly, in other groups, the number of *Lactobacillus* in male rats was significantly higher than that in females except for the Med group and Ant group after 1 week of drug intervention and the Low group, Med group, and Hig group after 3 weeks of drug intervention (Figure 8(d)).

**Figure 9:**
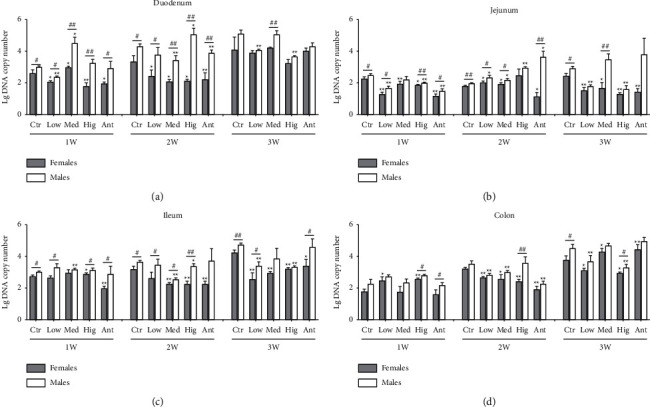
Effect of *S. flavescens* on the number of *Clostridium*, in various segments of rats with different genders. (a–d) Changes in the number of *Clostridium* in duodenum, jejunum, ileum, and colon after 1, 2, and 3 weeks of drug intervention, respectively. Ctr, Low, Med, Hig, and Ant represent normal group, low-, medium-, and high-dose groups of *S. flavescens* and antibiotic group, respectively. 1, 2, and 3 represent the rats for 1, 2, and 3 weeks of continuous drug intervention. The data was shown as means ± s.e.m, *n* ≥ 3, and Student's *t*-tests were used to generate the *P* values. *t*-test: ^*∗*^, *P* < 0.05; ^*∗∗*^, *P* < 0.01 versus Ctr; ^#^*P* < 0.05; ^##^*P* < 0.01 females versus males.

For *Clostridium*, in duodenum: after continuous drug intervention, compared with the Ctr group, the number of *Clostridium* in female rats from Low group or Hig group and in female and male rats from Ant group decreased first and then returned to normal. In Med group, the number of *Clostridium* both in male and female rats firstly increased, then decreased, and finally returned to normal. The number of *Clostridium* in male rats was significantly decreased in Low group, and increased first and then decreased in Hig group. In terms of gender, in addition, after 1 and 2 weeks of drug intervention, the number of *Clostridium* in male rats was significantly higher than that in females among each group. The number of *Clostridium* in male rats was significantly higher than that in females from the Med group after 3 weeks of drug intervention (Figure 9(a)). In jejunum: after continuous drug intervention, compared with the Ctr group, the number of *Clostridium* both in female rats of the Low group and Med group and males of Low group and Hig group decreased first, then increased and finally decreased, while the number of *Clostridium* in female rats of the Hig group decreased significantly after 2 weeks of drug intervention. Interestingly, except for the Med group after 1 weeks of drug intervention, the Hig group after 2 and 3 weeks of drug intervention, and Low group and Ant group after 3 weeks of drug intervention, in other groups, the number of *Clostridium* in male rats was significantly higher than that in females (Figure 9(b)). In ileum: after continuous drug intervention, compared with the Ctr group, in Low group, the number of *Clostridium* both in female and male rats decreased significantly 3 weeks after drug administration, while the number of *Clostridium* was significantly decreased both in female rats of the Med group or Ant group and males of the Hig group. Moreover, in other groups, the number of *Clostridium* in male rats was significantly higher than that in females except for the Med group after 1 and 3 weeks of drug intervention, Ant group after 2 weeks of drug intervention, and Hig group after 3 weeks of drug intervention (Figure 9(c)). In colon: after continuous drug intervention, compared with the Ctr group, the number of *Clostridium* in female rats in each group decreased first and then increased. The number of *Clostridium* in male rats of the Low group was significantly decreased, and that of male rats of the Hig group was significantly decreased 3 weeks after administration, while the number of *Clostridium* in male rats of the Med group and Ant group was firstly decreased and then returned to normal. Furthermore, the number of *Clostridium* in male rats was significantly higher than that in females in the Hig group after 1, 2, and 3 weeks of drug intervention, in the Ant group after 1 week of drug intervention and in the Ctr group after 3 weeks of drug intervention (Figure 9(d)).

All in all, these results suggested that *S. flavescens* has different effects on microflora in different intestinal segments, and the effect had obvious dose-time (dosing dose and duration of dosing) regularity and gender difference.

## 4. Discussion

Sexual dimorphism is a frequent feature for a variety of common disorders, including metabolic, cardiovascular, psychiatric, and autoimmune diseases [25–27]. A large amount of data has shown that the gut microbiome is closely related to many of these disorders, but the exact mechanisms that mediate these associations are poorly understood. Therefore, it is necessary to understand some of the factors that influence gut microbiota, including host diet, medications, age, pets, environment, and heredity [28–33]. In recent years, some studies suggest that gender has no or very limited impact on the gut microbiota [34, 35], while others have shown that there are differences in the composition of gut microbiome between sexes, but the mechanism that causes the differences remains unclear [7, 8, 36, 37]. Thus, we aimed to examine sex differences in gut microbiota composition of fecal and jejunal samples. Interestingly, our results show significant sex differences in gut microbiota composition, especially in the jejunum, and while this difference was not significant in fecal sample (Figure 1), which may be due to other factors affecting the structure of intestinal flora in different intestinal segments.

In modern clinical studies, intestinal flora is always represented by colon or fecal flora, mainly because it is difficult to obtain the flora samples of human small intestine and the microbial quantity of small intestine is significantly lower than that of large intestine [38]. However, the composition and quantity of the gut microbiota change along the longitudinal axis of the intestinal tract, and the flora of each intestine segment plays different functions [39]. Therefore, it is of practical significance to explore the differences of intestinal flora in different parts of the intestinal tract and the changes after drug intervention. The gastrointestinal tract is the main place where *S. flavescens* works. This study suggested that the low dose of *S. flavescens* had little effect on the microflora structure of ileum and colon, especially in male rats, which might be the reason for the insufficient dosage, except that there were gender differences in the effects of *S. flavescens* on the microflora composition of each intestinal segment of rats (Figures 2–5).

### 4.1. Effects of *S. flavescens* on the Growth of *Enterococcus* in Different Intestinal Segments

*Enterococcus* is a kind of Gram-positive bacteria widely existing in the human gastrointestinal tract. It does not harm human health under normal circumstances. However, when the intestinal barrier is damaged due to the disturbance of gut microbiota, *Enterococcus* will break through the intestinal barrier and cause urinary tract infection, bacteremia, intraperitoneal infection, and endocarditis [40]. Here, after *S. flavescens* treatment, the number of *Enterococcus* in duodenum and colon of rats showed a trend of first increasing and then decreasing (Figure 6), suggesting that *S. flavescen*s has potential anti-*Enterococcus* effect. In addition, lincomycin promoted the growth of *Enterococcus* in the jejunum, ileum, and colon and inhibited the growth of *Enterococcus* in the duodenum (Figure 6). This is related to the special resistance of *Enterococcus* to lincoamide antibiotics [41]. However, *Enterococcus* is resistant to many disinfectants and extreme environment in vitro and has low sensitivity to many antibiotics in vivo. Therefore, this result provides some ideas for finding drugs to inhibit resistant *Enterococcus* [41]. Interestingly, after 3 weeks of treatment, low and high doses of *S. flavescens* inhibited and promoted the growth of *Enterococcus*, respectively (Figure 6). This phenomenon may be caused by the bidirectional regulation in the efficacy of TCM, which often shows multiple effects due to the diversity of its components, the content difference of each component, and the different dose of administration. Moreover, for a single component, its efficacy increases within a certain range with the increase of the dose of administration. However, due to the complex and diverse components of TCM, the forms of enhancing its efficacy may be different, such as the increase of one pharmacological effect and the decrease of the other, or the neutralization of the two pharmacological effects [42]. Therefore, TCM often produces the effect of low-dose and high-dose opposite phenomenon. For example, two saponins in *Panax notoginseng* can inhibit and promote platelet aggregation, respectively [43, 44]. Lung cancer is characterized by sexual dimorphism [45], and *Enterococcus* is closely associated with lung cancer as a potential marker [46] The results of this study showed that *S. flavescens* was more beneficial to *Enterococcus* growth in male rats than in females, suggesting that the anti-lung cancer effect of *S. flavescens* might be more obvious in females.

### 4.2. Effects of *S. flavescens* on the Growth of *Bacteriodes* in Different Intestinal Segments

*Bacteriod*es are obligate anaerobic Gram-negative bacteria, which are significantly enriched in human colon [47]. It is generally accepted that *Bacteriodes* are probiotics that are helpful to body health, mainly manifested in (1) providing nutrition and energy for the host through the degradation of short-chain fatty acids produced by polysaccharides, (2) controlling intestinal lipopolysaccharide (LPS) and alleviating the inflammatory response of the body, (3) providing nutrition or antagonizing other bacterial communities to stabilize the intestinal microecological balance [48, 49]. In this study, after 1–3 weeks of *S. flavescens* intervention, the number of *Bacteroides* in each intestinal segment of rats showed a certain decreasing trend (Figure 7), which may be related to the intervention of *S. flavescens* in type 2 diabetes. Shao et al. [50] found that the abundance of *Bacteriodes* in rat fecal stool was significantly reduced after the intervention of *S. flavescens* extract in rats with type 2 diabetes. Estrogen is negatively correlated with the incidence of type 2 diabetes, that is, type 2 diabetes is associated with gender [51]. Here, in this study, it was found that *S. flavescens* could inhibit the growth of *Bacteroides* to a greater extent in female rats than in males (Figure 7), suggesting that *S. flavescens* could intervene in type 2 diabetes, and the intervention was gender-specific.

### 4.3. Effects of *S. flavescens* on the Growth of *Lactobacillus* in Different Intestinal Segments

*Lactobacillus*, a group of Gram-positive bacteria belonging to Firmicutes, is widely distributed in nature, animals, and human bodies and play a role in metabolizing carbohydrates to produce lactic acid [52]. Although the proportion of *Lactobacillus* in human intestine is low, it has strong stability and is closely related to human health [53, 54]. The main functions of *Lactobacillus* as probiotics include synthesizing vitamins needed by the body, antagonizing pathogenic bacteria, and enhancing intestinal barrier function and immune effect [55]. In this study, it was found that after 1 week of lincomycin intervention, the number of *Lactobacillus* in all intestinal segments of both sexes in rats decreased significantly but gradually recovered after 2 or 3 weeks of intervention (Figure 8), which may be due to the fact that lincomycin played a full bacteriostatic role in the early stage of intervention, but with the increase of time of drug intervention, the reduction of antagonistic bacteria or the development of drug resistance of *Lactobacillus* led to the recovery of the population. Long-term use of bitter and cold TCM can cause gastrointestinal tract injury. *Gardenia jasminoides* and *S. flavescens* are one of the bitter and cold TCM. Large dose of *Gardenia jasminoides* induced intestinal injury in rats through oral administration, causing severe diarrhea, weight loss, intestinal tissue morphological damage, and other symptoms [56]. This study showed that the number of *Lactobacillus* increased in the duodenum and jejunum of rats after 2 weeks of *S. flavescens* intervention, and the results were opposite in the ileum and colon, while after 3 weeks of *S. flavescens* intervention, the number of *Lactobacillus* has decreased to varying degrees in the duodenum and ileum of rats, the number of *Lactobacillus* in the jejunum has increased, and the number of *Lactobacillus* in the colon has returned to normal levels (Figure 8). These results suggest that *S. flavescen*s can damage the small intestine and relieve ulcerative colitis [57]. In particular, the effect of low doses of *S. flavescens* on the number of Lactobacillus was greater. Immune response reflects sex-specificity. It is generally believed that estrogen produces proinflammatory effects while androgens are anti-inflammatory [58]. In this study, compared with male rats, the number of *Lactobacillus* in the colon of females increased after 3 weeks of intervention by *S. flavescens* (Figure 8), revealing the prominent role of *S. flavescens* in preventing ulcerative colitis in females.

### 4.4. Effects of *S. flavescens* on the Growth of *Clostridium* in Different Intestinal Segments

*Clostridium* is a Gram-positive anaerobe that produces spores and is the most common pathogen in antibiotic-associated diarrhea [59]. Shao et al. [60] found that the number of *Clostridium* in the intestinal tract of antibiotic-associated diarrhea model mice established by gentamicin and cefradine was significantly increased. Lincomycin has also been used as an inducer for antibiotic-associated diarrhea [61]. Lincomycin dose used in this study first reduced and then recovered the number of intestinal *Clostridium* in rats 1–3 weeks after intervention (Figure 9); *Clostridium* produces many protein toxins, often causing neurotoxicity, tissue toxicity, intestinal infection, etc. [62]. Here, except for male rats with medium dose of *S. flavescens*, after 3 weeks of *S. flavescens* intervention, the number of *Clostridium* of other groups in all intestinal segments was significantly decreased, and the effects were different between sexes (Figure 9). The results suggested that *Clostridium* is a potential target of *S. flavescens* against intestinal inflammation, and it also provides some ideas for *S. flavescens* to interfere with antibiotic-associated diarrhea.

In summary, in this study, it was found that the effects of *S. flavescens* on the growth of bacteria is diverse in different intestinal segments, which may be caused by multiple factors. Firstly, the pH value of intestines has a great influence on drug absorption and metabolism and increases gradually from the small intestine to the large intestine [63]. Different pH values in different intestinal segments perhaps lead to different metabolic degree of *S. flavescens*, thus affecting its effect on the growth of intestinal flora in each intestinal segment. Secondly, intestinal flora is widely distributed in various areas of the intestine, and the number or species of intestinal flora vary significantly due to different physiological environments in different intestinal segments such as pH value, intestinal lumen contents, and oxygen content [64, 65]. However, the interaction between drugs and intestinal flora is usually reciprocal; thus, drugs can affect the quantity and structure of intestinal flora while intestinal flora can also affect the drug effect [31]. The different intestinal flora resulted in the inconsistency of the interaction between intestinal flora and *S. flavescens* in different intestinal segments. In addition, with drug absorption by intestine and drug metabolism by intestinal flora, the effective concentration of oral drugs decreases from small intestine to large intestine [66, 67]. This may be one of the reasons for the different effects of *S. flavescens* on the growth of intestinal flora in different intestinal segments. Most modern studies focus on the effects of drugs on all the flora in the whole intestinal tract, but ignore the differences in the effects of drugs on the flora in different parts of the longitudinal axis of the intestinal tract. This study proved that the number of four dominant bacteria in different intestinal segments of rats was different and then found that under the intervention of *S. flavescens*, the changes of four dominant bacteria in different intestinal segments were different (Figures 6–9). These results laid a foundation for the study of the prevention and treatment of intestinal microbiota-related diseases by *S. flavescens*. It also provides some new directions for the role of *S. flavescens* in intestinal diseases such as colitis and intestinal cancer [68, 69].

## 5. Conclusion

In SD rats, there were gender differences in the composition of gut microbiota, especially in the jejunum. Besides, the effects of *S. flavescens* on the gut microbiota of various intestinal segment were different between genders under different dosages and different continuous administration times. These results provides a certain experimental basis for the clinical application of *S. flavescens*, especially for the prevention and treatment of diseases targeting the gut microbiota, and provides some ideas for the drug use difference of *S. flavescens* and other TCM targeting different genders.

## Data Availability

The raw data of the qRT-PCR was uploaded in the supplementary. The sequence data used to support the findings of this study have been deposited to the NCBI Sequence Read Archive under the BioProject accession number (NCBI SRA number: PRJNA807458).

## Supplementary Materials

Supplementary Materials Table S1, S2: analysis of similarity (ANOSIM) and permutational multivariate analysis of variance (PERMANOVA) of gut microbiota composition in SD rats, respectively. Table S3: the influence of sex on the structure of intestinal microflora. Figure S1: relative abundances distributions per group for the taxa (at phylum level) in SD rats.
