# Supplementary material for: Sex Difference of Effect of Sophora flavescens on Gut Microbiota in Rats
Source: Evid Based Complement Alternat Med. 2022 Mar 15;2022:4552904. doi: 10.1155/2022/4552904 (PMC8941563; doi:10.1155/2022/4552904)
Supplement: Supplementary Materials — Table S1, S2: analysis of similarity (ANOSIM) and permutational multivariate analysis of variance (PERMANOVA) of gut microbiota composition in SD rats, respectively. Table S3: the influence of sex on the structure of intestinal microflora. Figure S1: relative abundances distributions per group for the taxa (at phylum level) in SD rats. [file 4552904.f1.zip › 4552904.f1/Supplementary data.pdf]

**Table S1 Analysis of similarity (ANOSIM) of gut microbiota composition in SD rats**

| Groups | R | *P*.value | Dissimilarity | Permutation |
|---|---|---|---|---|
| Females-F VS Males-F | 0.14 | 0.06 | Bray-curtis | 999 |
| Females-J VS Males-J | 0.25 | 0.02 | Bray-curtis | 999 |

F stands for fecal samples; J stands for jejunal samples, n=10 per group

**Table S2 Permutational multivariate analysis of variance (PERMANOVA) of gut microbiota composition in SD rats.**

| Groups | Df | SumsOfSqs | F.Model | R2 | *P*.value |
|---|---|---|---|---|---|
| Females-F VS Males-F | 1 | 0.29 | 1.95 | 0.10 | 0.049 |
| Females-J VS Males-J | 1 | 0.63 | 3.07 | 0.15 | 0.02 |

F stands for fecal samples; J stands for jejunal samples, n=10 per group.

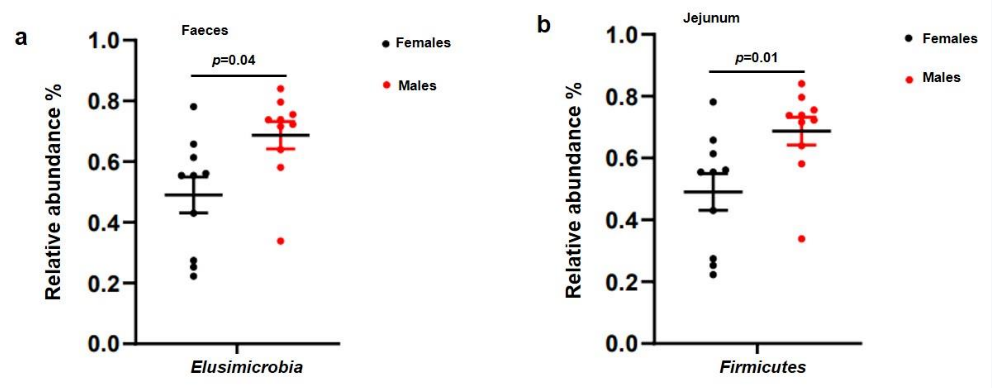


Fig. S1 Relative abundances distributions per group for the taxa (at phylum level). In faeces or jejunum, the individuals are grouped by Females, Males, n=10 per group, *P* values from Mann–Whitney U test. .shown for the bacterial taxonomic rank of phylum.

**Table S3 The influence of sex on the structure of intestinal microflora**

| Samples | Taxonomy (Family or Genus) | Relative abundance (%, Mean values±SD, n=10) | | *p*-value (adjusted)* |
|---|---|---|---|---|
| | | Females | Males | |
| Faeces | *Alloprevotella* | **0.04±0.04** | 0.01±0.04 | 0.002 |
| | *Elusimicrobium* | 0.01±0.01 | **0.030±0.01** | 0.04 |
| | *Helicobacter* | **0.01±0.01** | 0.001±0.001 | 0.01 |
| | *Lachnospiracea_incertae_sedis* | 0.001±0.001 | **0.01±0.003** | 0.0005 |
| | *Desulfovibrionaceae* | **0.003±0.003** | 0.001±0.001 | 0.02 |
| *Jejunum* | *Anaerorhabdus* | 0.002+0.002 | **0.01±0.004** | 0.03 |
| | *Blautia* | 0.05±0.06 | **0.12±0.10** | 0.04 |
| | *Corynebacterium* | 0.002+0.005 | **0.01±0.01** | 0.02 |
| | *Roseburia* | **0.01±0.02** | 0.0002±0.0003 | 0.01 |
| | *Rothia* | **0.32±0.24** | 0.10±0.16 | 0.01 |
| | *Lachnospiraceae* | 0.005±0.006 | **0.01±0.01** | 0.04 |
| | *Coriobacteriaceae* | 0.007±0.01 | **0.03±0.03** | 0.02 |

* *p* value were adjusted using Benjamini-Hochberg FDR
